# Experimental comparison of photon versus particle computed tomography to predict tissue relative stopping powers

**DOI:** 10.1002/mp.15283

**Published:** 2021-11-16

**Authors:** Esther Bär, Lennart Volz, Charles‐Antoine Collins‐Fekete, Stephan Brons, Armin Runz, Reinhard Wilhelm Schulte, Joao Seco

**Affiliations:** ^1^ Department of Medical Physics and Biomedical Engineering University College London London UK; ^2^ Department of Radiotherapy Physics University College London Hospitals NHS Foundation Trust, Radiotherapy Physics London UK; ^3^ Biomedical Physics in Radiation Oncology German Cancer Research Center (DKFZ) Im Neuenheimer Feld Heidelberg Germany; ^4^ Heidelberg Ion Beam Therapy Center Im Neuenheimer Feld Heidelberg Germany; ^5^ Medical Physics in Radiation Oncology German Cancer Research Center (DKFZ) Im Neuenheimer Feld Heidelberg Germany; ^6^ Division of Biomedical Engineering Sciences Loma Linda University Loma Linda California USA; ^7^ Department of Physics and Astronomy Heidelberg University Germany

**Keywords:** dual‐energy computed tomography, particle computed tomography, proton stopping power

## Abstract

**Purpose**: Measurements comparing relative stopping power (RSP) accuracy of state‐of‐the‐art systems representing single‐energy and dual‐energy computed tomography (SECT/DECT) with proton CT (pCT) and helium CT (HeCT) in biological tissue samples.

**Methods**: We used 16 porcine and bovine samples of various tissue types and water, covering an RSP range from 0.90±0.06 to 1.78 ±0.05. Samples were packed and sealed into 3D‐printed cylinders (d=2 cm, h=5 cm) and inserted into an in‐house designed cylindrical polymethyl methacrylate (PMMA) phantom (d=10 cm, h=10 cm). We scanned the phantom in a commercial SECT and DECT (120 kV; 100  and 140 kV/Sn (tin‐filtered)); and acquired pCT and HeCT (E∼200 MeV/u, 2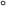 steps, $∼6.2\times 10^{6}$ (p)/$∼2.3\times 10^{6}$ (He) particles/projection) with a particle imaging prototype. RSP maps were calculated from SECT/DECT using stoichiometric methods and from pCT/HeCT using the DROP‐TVS algorithm. We estimated the average RSP of each tissue per modality in cylindrical volumes of interest and compared it to ground truth RSP taken from peak‐detection measurements.

**Results**: Throughout all samples, we observe the following root‐mean‐squared RSP prediction errors ± combined uncertainty from reference measurement and imaging: SECT 3.10±2.88%, DECT 0.75±2.80%, pCT 1.19± 2.81%, and HeCT 0.78±2.81%. The largest mean errors ± combined uncertainty per modality are SECT 8.22 ±2.79% in cortical bone, DECT 1.74±2.00% in back fat, pCT 1.80 ±4.27% in bone marrow, and HeCT 1.37±4.25% in bone marrow. Ring artifacts were observed in both pCT and HeCT reconstructions, imposing a systematic shift to predicted RSPs.

**Conclusion**: Comparing state‐of‐the‐art SECT/DECT technology and a pCT/HeCT prototype, DECT provided the most accurate RSP prediction, closely followed by particle imaging. The novel modalities pCT and HeCT have the potential to further improve on RSP accuracies with work focusing on the origin and correction of ring artifacts. Future work will study accuracy of proton treatment plans using RSP maps from investigated imaging modalities.

## 1 INTRODUCTION

For particle therapy treatment planning, three‐dimensional (3D) maps of the relative stopping powers (RSPs) (stopping power relative to water) of the patient's tissues are needed for treatment simulation of range and dose calculation. In current clinical practice, the RSP values for treatment planning are estimated from a conventional single‐energy computed tomography (SECT) scan. The SECT images acquired for treatment simulation are used to estimate RSP values with an empirical multilinear calibration curve. These calibration curves can be derived in two different ways, based on either tissue‐equivalent materials or a list of standardized human tissues.^1^ Because the relation between CT numbers and RSP values is not bijective, the so estimated RSP values can be erroneous, leading to uncertainties in the resulting treatment plan. Range uncertainties of up to 1.8% were shown to be introduced through this procedure.^2^, ^3^

In recent years, dual‐energy computed tomography (DECT) gained traction as an imaging modality for proton radiotherapy (RT) treatment planning. Several algorithms were developed to estimate tissue parameters from DECT^4^, ^5^, ^6^, ^7^, ^8^, ^9^, ^10^, ^11^, ^12^, ^13^ to estimate the RSPs of tissues. It was shown in simulations and experimental studies that DECT can be more accurate than SECT in estimating the RSPs. A reduction in range uncertainties to about 1% can be achieved,^14^, ^15^, ^16^, ^17^, ^18^, ^19^ at the cost of an increased noise level for a fixed dose.

A more direct way to measure the RSP values is particle imaging. Particle imaging, predominantly performed with protons (proton CT; pCT), measures the energy loss of charged particles after traversing the object. This energy loss is directly linked to the path integral over the RSP, enabling RSP reconstruction without the need of an empirical calibration between physically unrelated quantities. In recent experimental investigations using prototype detector development and tissue equivalent plastic samples, pCT RSP accuracy has been shown to be on par with DECT.^20^ At the same time, pCT shows a distinct dose advantage compared to SECT and DECT, due to the low proton fluence required, and the fact that only the proton entrance dose lies within the patient.^21^, ^22^, ^23^ Yet, due to the charged nature of protons, they suffer multiple Coulomb scattering as they cross a medium and produce a blurred image, potentially causing related misrepresentation of the RSP for small features^24^, ^25^ and the underestimation of range mixing effects.^26^

In recent studies, helium ion CT (HeCT) has been identified as a potential method for high accuracy RSP. Helium particles scatter less due to their lower charge to mass ratio and should, therefore, produce sharper images.^24^, ^27^, ^28^, ^29^ HeCT could hence be a viable choice to keep the high RSP accuracy and mostly low dose promised by proton imaging while providing a clinically suitable spatial resolution and relatively low fragmentation.^23^ Still, helium beams are rare and helium imaging is not accessible by most treatment centers globally.

Thus, every modality suffers from limitations, whether it is the lack of a bijective relation in the required calibration (SECT), the noise introduced by extracting twice the amount of information from a fixed dose (DECT),^30^, ^31^ the lack of spatial resolution in clinical images (pCT), or the comparably limited availability for treatment planning (HeCT). Therefore, an unbiased comparison is required to compare these modalities' capacities to produce accurate RSP maps. Dedes et al.^20^ recently performed such a comparison on plastic inserts between pCT and DECT, observing a slight benefit for pCT in terms of RSP accuracy. However, the study was limited to plastic materials, which could bias the photon CT results depending on the way they are calibrated.

The purpose of this work is to perform a comparison of the RSP accuracy provided by photon and particle CT modalities based on available systems. We use 16 postmortem porcine and bovine tissue samples and water. The samples were inserted into a custom‐built cylindrical phantom to acquire RSP maps from SECT and DECT using a dual‐source dual‐energy CT scanner and pCT and HeCT using the US pCT collaboration prototype scanner, operated in the scope of the experiment at the Heidelberg Ion‐Beam Therapy Center (HIT). The results are compared to RSP reference values acquired using peak detection measurements.

## 2 MATERIALS AND METHODS

We compared four competing modalities acquired with their current best detection system, namely HeCT, pCT, SECT, and DECT, concerning their capacities to produce voxelized RSP values for animal tissue samples. In the context of particle therapy treatment planning, RSP precision and accuracy are paramount and the metrics used for comparison. To minimize uncertainties and enact a fair comparison across all modalities, we developed a bespoke phantom for this study (Section 2.1). To provide clinically relevant results, we studied fresh bovine and porcine tissues sample as they are more closely representative of human tissues (Section 2.2). To provide a basis for comparison, we acquired a reference RSP for each tissue by performing Bragg‐peak shift measurements (Section 2.3). Both single‐energy and dual‐energy CT images were acquired and calibrated to RSP maps (Section 2.4.1 and Section 2.4.2, respectively). Helium and proton CT tomography images were acquired using the same phantom and tissues (Section 2.5).

### 2.1 Phantom

A custom‐built phantom is used in this study to hold the tissue samples. The phantom consists of a cylindrical polymethyl methacrylate (PMMA) body (15 cm diameter, 10 cm height) with five cylindrical cavities of 2 cm diameter, positioned at different radial distance to the center (one at 3 cm, one at 5 cm, and two at 4 cm distance). The schematic of the phantom is shown in Figure 1a. The phantom is designed to house 10 cylindrical sample containers, each 2 cm in outer diameter and 5 cm in height. The sample containers were 3D‐printed with wall/lid thickness of 1 mm and made of VeroClear epoxy (Sculpteo, Villejuif, France). The 3D‐printer slice thickness was 16μm, which results in an uncertainty of 0.008 mm on the thickness of the lid/the cylinder height.

**FIGURE 1:**
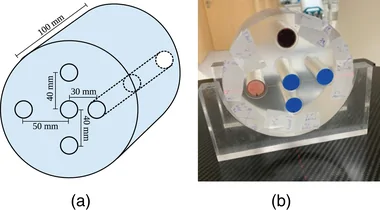
(a) Diagram showing the dimension of the cylindrical PMMA phantom alongside with insert holes. (b) Picture showing the developed phantom filled with fresh porcine and bovine tissues.

### 2.2 Sample preparation

To prepare the fresh tissue samples, we collected porcine and bovine tissues from the local butcher. A total of 16 tissue samples were collected. From the pig, we collected lung, belly fat, back fat, blood, cheek muscle, two samples of loin muscle, brain, two samples of kidney, two samples of liver, and trabecular bone, whereas from the cow we collected bone marrow, leg muscle, and cortical bone. Soft tissues were cut into pieces and filled and pressed into the containers. Trabecular and cortical bone pieces were taken from a single piece of bone, sawn into shape and then finely sanded down to exactly match the length of the container, then filled in. One cylinder was left empty to measure the water‐equivalent thickness (WET) of the container wall. Another container was filled with distilled water. After the samples were filled in, the sample containers were sealed using the same epoxy as used for 3D printing to isolate the tissue samples and prevent drying. All samples were kept in the fridge in between preparation and measurements to avoid decay, and all measurements were performed within 24 h of sample preparation: Samples were prepared in the morning, photon CT scans were acquired in the afternoon, and range reference measurements and particle CT modalities were acquired in the evening or at night. To observe potential decay effects, a second set of SECT/DECT scans was acquired on the day after the measurements. Minor air enclosures were present in some of the samples and could not be avoided during sample preparation.

### 2.3 Reference RSP acquisition

All experiments using particle beams were performed at the HIT. To obtain a reference value for the RSP, we performed peak detection measurements using the PeakFinder (PTW, Freiburg, Germany) variable water column in a clinical carbon beam along the insert axial direction; see Figure 2a for a photograph of the setup. A carbon beam was chosen due to the fine available beam spot size at HIT, which paired with the carbon ions' low scattering and straggling properties provide a precise estimate of the sample WET. The RSP is only weakly depending on the ion type in the chosen energy range, such that the use of pCT or HeCT to infer the RSP of a carbon beam can be justified. Moreover, a previous study on pCT^32^ has compared reconstructed RSPs for plastic inserts to carbon beam measured RSPs with high accuracy. The beam range was chosen to be slightly larger than the WET of the tissue sample to keep range straggling at a minimum (*E* = 200.3 MeV/u for soft tissues, 250.1 MeV/u for bone tissues; beam width = 6.7  and 4.3 mm (full width at half maximum), respectively). For each sample and the empty container, the peak position was measured in steps of 0.1 mm; interpolation between measurement points was done using a spline interpolation method. From the interpolated peak, the 80% proximal and distal dose position was found for all samples and the empty container (see Figure 2b). The WET of the samples (excluding the container) was then calculated as the difference in the distal positions of the 80% dose ($R_{80}$) of the empty container and the sample

(1) $${\mathrm{WET}}_{i}=R_{80,\mathrm{empty}}-R_{80,i},$$

with i=(1,2,…17) representing the tissue samples and water. The average RSP of each sample is calculated as

(2) $${\mathrm{RSP}}_{\text{ref},i}=\frac{{\mathrm{WET}}_{i}}{t},$$

where t is the sample thickness of 48 mm. The average RSP values served as reference RSPs in this study. Uncertainties for these reference measurements were estimated by establishing the combined uncertainty imposed on the measurement by peak widening due to natural energy straggling and the presence of the heterogeneous sample. To estimate the reference uncertainties, we separate the effect of straggling in a homogeneous medium from the peak widening introduced by a heterogeneous sample. Particles in a homogeneous medium experience energy straggling which naturally leads to a broadening of the Bragg peak. Slight sample inhomogeneity causes additional variability in the particles' energy and thus to additional broadening of the peak. We consider energy straggling in a homogeneous medium as the width of the Bragg peak without a container or sample in place ($w_{\mathrm{hom}}$). It follows that the peak width measured with sample i in place ($w_{\text{het},i}$) represents the combined uncertainty of the sample i. Then, we can assume the degree of sample heterogeneity as the difference in measured peak widths $w_{\mathrm{hom}}$ and $w_{\text{het},i}$, and the RSP variability is expressed as

(3) $$\sigma_{\text{ref},i}=\frac{\sqrt{w_{\text{het},i}^{2}-w_{\mathrm{hom}}^{2}}}{t},$$

where *t* is the sample thickness. The terms in the subtraction are squared because the underlying processes which lead to the peak broadening, namely energy straggling and heterogeneity‐induced straggling, are assumed to be statistically independent.

**FIGURE 2:**
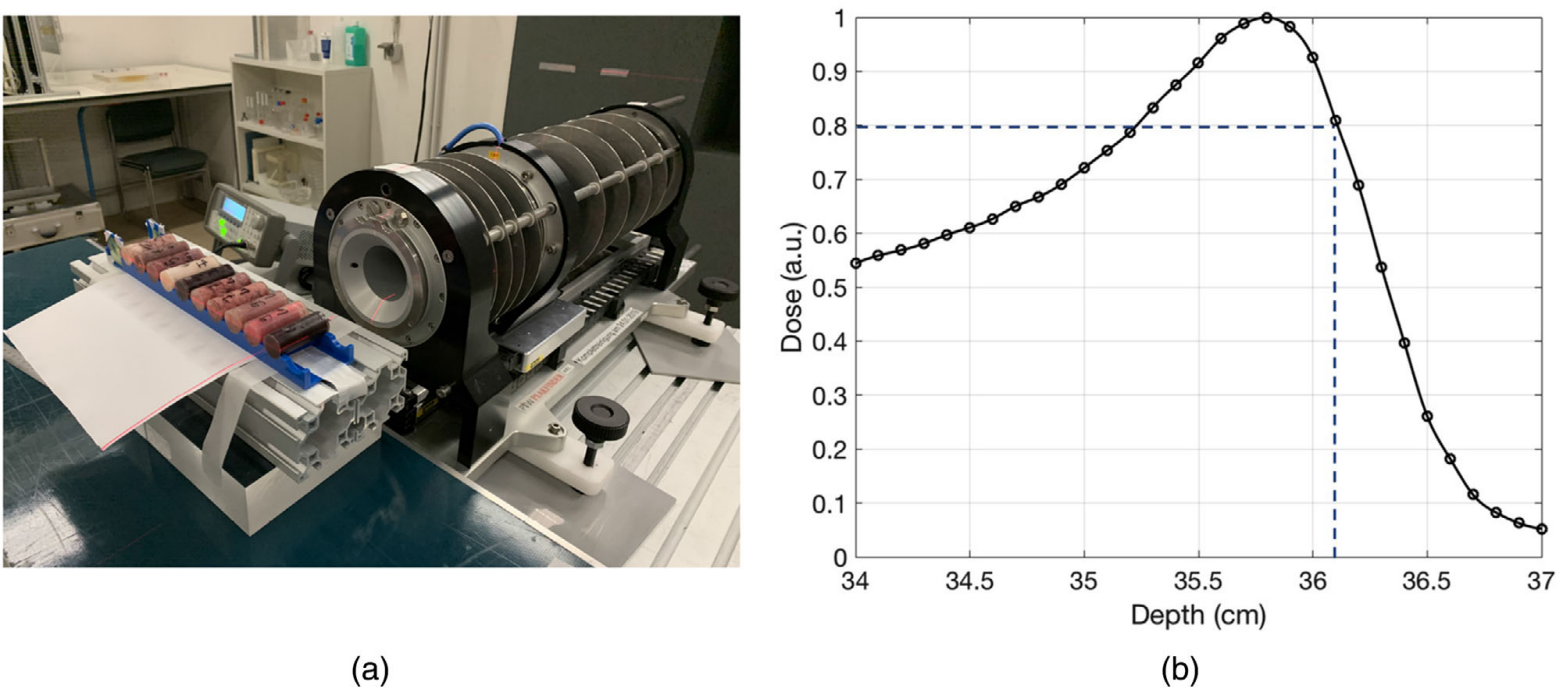
Pictures showing the measurement setups for (a) reference measurement using the PeakFinder and (b) an exemplary measured Bragg peak curve, with a dotted line representing the positions of the 80% distal dose (R80). The samples were placed on a motorized stage to accelerate measurement and improve precision.

### 2.4 Photon CT acquisition and RSP prediction

#### 2.4.1 Single‐energy CT

All CT images were obtained at the German Cancer Research Center (DKFZ) using a Somatom Definition Flash dual‐source scanner (Siemens Healthineers, Erlangen, Germany). To obtain single‐energy CT images, we used a tube voltage of 120 kV, a tube current (in terms of exposure time product) of 215 mAs, and a field of view of 500 mm (CTDIvol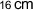 of 59.8 mGy, acquisition time: 5 s). Reconstruction was done using a H30s filtered back projection reconstruction kernel with a slice thickness of 2 mm and a pixel size of 0.977 × 0.977 mm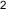. For the CT to RSP conversion, we scanned the Gammex RMI 467 electron density phantom (SunNuclear, Melbourne, FL, USA) with tissue‐equivalent materials, densities, mean excitation energies (I‐values), and theoretically calculated RSP values as listed in Table 1. Electron densities were obtained from the densities and elemental compositions of the materials given by the manufacturer. I‐values were calculated using the Bragg additivity rule from elemental compositions and elemental I‐values taken from Bär et al.^33^ RSP values were calculated using the Bethe formula^34^ with protons (*E* = 195 MeV) as projectile.

**TABLE 1:** List of relative electron densities, I‐values, and theoretically calculated RSP of the materials used to calibrate the SECT and DECT stoichiometric methods

Material	$\rho_{e}$	I‐value	RSP
Water	1.00	78.73	1.00
LN450 lung	0.44	77.47	0.44
AP6 adipose	0.93	67.07	0.94
BR12 breast	0.97	68.73	0.98
CT solid water	0.99	71.00	1.00
LV1 liver	1.06	71.00	1.07
SR2 brain	1.04	66.85	1.06
Muscle	1.02	71.01	1.03
CB2 ‐ 30% CaCO 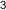	1.27	86.51	1.25
CB2 ‐ 50% CaCO 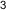	1.46	99.95	1.42
SB3 cortical bone	1.68	113.79	1.61
B200 mineral bone	1.11	82.51	1.10
IB3 inner bone	1.10	82.41	1.09

To estimate RSP values from SECT images, the stoichiometric calibration by Schneider et al. was used.^1^ We first performed the energy calibration using equation (8) in Schneider et al. to find the energy‐dependent coefficients $K^{ph}$, $K^{coh}$, and $K^{KN}$ from the CT numbers (in Hounsfield units, HU) of the tissue‐equivalent materials. These coefficients were then used to calculate CT numbers for 70 tabulated human tissues.^35^, ^36^ We subsequently obtained the stoichiometric calibration curve by plotting the RSPs for these tissues against the calculated CT numbers and fitting a curve to these points. We combined four linear fits to cover the following regions: lung (HU < −150), adipose tissue (−150 ≤ HU < −30), soft tissues (−30 ≤ HU < 200), and bone tissues (200 ≤ HU). The tissue‐sample‐containing 3D‐printed containers were inserted into an in‐house designed cylindrical phantom made of PMMA material, as shown in Figure 3a. The SECT calibration curve is shown in Figure 3b,c. The RSPs are estimated voxelwise from the CT numbers using the stoichiometric calibration curve.

**FIGURE 3:**
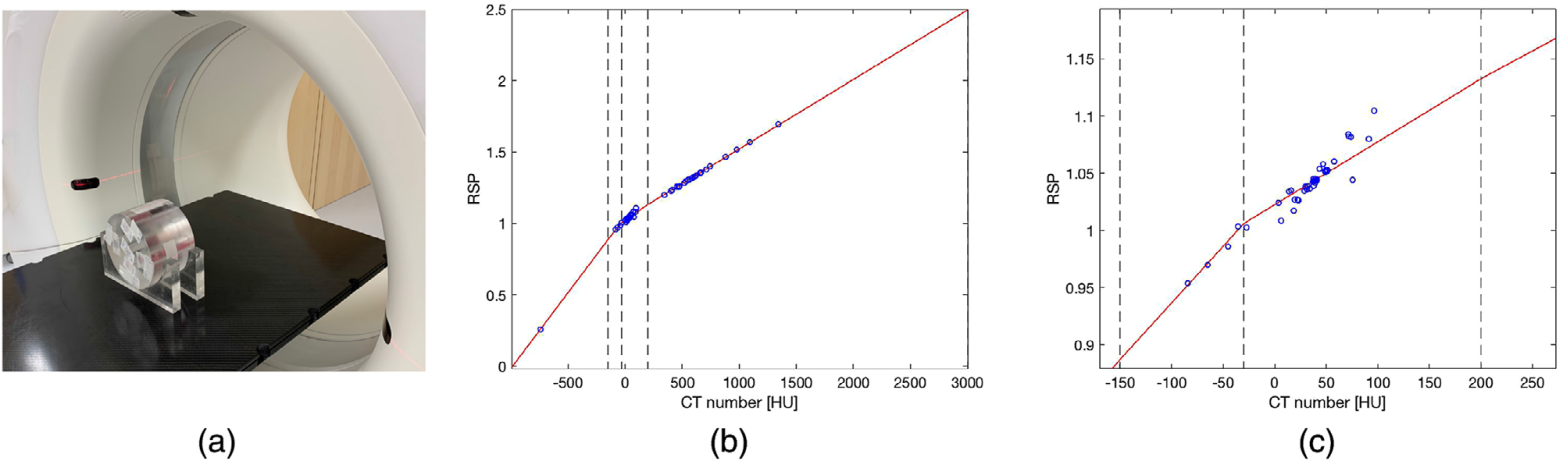
Illustration of the SECT stoichiometric calibration used in this work. (a) Cylindrical PMMA phantom centerd in the Somatom scanner. (b) Calibration curve over the whole CT range. The points represent the tabulated tissues, the solid line the fitted curve, and the dashed lines the fit regions. (c) Zoom into the soft tissue region.

#### 2.4.2 Dual‐energy CT

To obtain dual‐energy CT images, we used two tube voltages of, respectively, 100  and 140 kV, with the latter tin filtered, and a tube current (in terms of exposure time product) of 174 mAs for both voltages (CTDIvol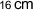 of 59.7 mGy, acquisition time: 17 s). Reconstruction was done using a D34s filtered back projection reconstruction kernel with a slice thickness of 2 mm and a pixel size of 0.977 × 0.977 mm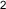. The DECT stoichiometric calibration by Bourque et al.^9^ was applied to estimate the RSPs from DECT images. This image‐based model uses two images acquired at different CT energies to estimate the relative electron density (RED) and effective atomic number (EAN) of the material on a voxel‐by‐voxel basis. From the DECT images, the dual‐energy ratio (DER) was calculated as the ratio of CT numbers taken from the low‐ and high‐energy images. The DER in a voxel was related to the EAN of the material within that voxel using a fifth degree polynomial. Similarly, a fifth degree polynomial relation was established between the CT number within a voxel and the RED. The parameters of the polynomials were found by least squares regression using a DECT scan of the Gammex RMI 467 electron density phantom with known elemental compositions and densities.

The EAN was used to estimate the I‐value per voxel via a parametric fit. The original paper from Bourque et al. suggests performing the parametric fit on a dataset of human reference tissues. We employed this approach with the sole modification that we used the elemental I‐values suggested in Bär et al.^33^ The REDs and I‐values estimated from a DECT scan were used to calculate the RSPs values voxelwise with the Bethe formula. We used an energy of 195 MeV and an I‐value of water of $I_{\mathrm{water}}=78.73$ eV as suggested by Bär et al.^33^

### 2.5 Particle CT acquisition and processing

To acquire state‐of‐the‐art particle CT scans, the particle CT scanner developed by the US pCT collaboration^37^, ^38^ was employed. It comprises two tracking detectors: one placed upstream (front tracker) and one downstream (rear tracker) of the phantom. Each of the tracking detectors consists of four planes of silicon strip sensors, recording the position and direction of each particle before and after the phantom. The tracker system is described in‐depth in Sadrozinski et al.^39^ The residual energy/range of each particle is measured with a five‐stage plastic scintillator energy/range detector that is described in detail in Bashkirov et al.^37^, and a schematic description is presented in Figure 4a.

**FIGURE 4:**
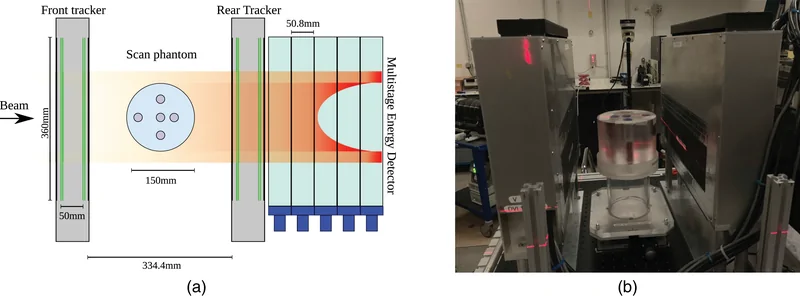
(a) Schematic representation of the cylindrical phantom placed inside the prototype particle CT scanner, along a sketch of the irradiation field and energy‐loss in the five‐stage energy detector as beam from light to dark red color. (b) The actual phantom filled with porcine and bovine tissue samples placed inside the pCT detector.

The US pCT collaboration prototype scanner was installed at the experimental cave of the HIT.^40^ Both helium ions and protons were used to generate particle CT images. The particles were delivered by raster scanning a field size of 20×10 cm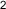 (H×V). The lateral spacing between adjacent beam spots was 3 mm. The field was delivered in a single spill of ∼4 s duration, and ∼4  s spill pause. The ion type specific beam parameters are given in Table 2. For the proton scan, the particle intensity was approximately twice that of the helium scan, due to vendor sensitivity limitations of the nozzle beam monitoring system: The scanning process relies on ionization chambers to ensure the spot dose is reached before moving to the next spot. These measure current, that is, particle rate times average energy deposit per particle. Hence, a factor 4 lower particle rate is possible for helium ions compared to protons at the same energy per nucleon before reaching the set sensitivity limit of the beam monitoring system.

**TABLE 2:** Beam parameters for proton and helium ions used to acquire the particle CT scans at the HIT center. The energy is given as the nominal value before the beam monitoring system

Particle	Protons	Helium ions
Energy (MeV/u)	200.11	200.38
Spot FWHM (mm)	12.6	10.2
Particle rate (MHz)	∼1.35	∼0.7
Approximate number of particles		
per projection	$∼6.2\times 10^{6}$	$∼2.3\times 10^{6}$
per projection per mm 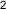	310	115
Total used after filtering	$∼4.2\times 10^{8}$	$∼8\times 10^{7}$

It is important to note that the pCT prototype's intended use is with a broad beam at particle rates up to 1.2 MHz. The use of pencil beam scanning at high rates leads to increased pileup in the tracker and energy detector (only due to the high rate) of the system which impairs the overall dose efficiency.^38^ Further details on the beam quality are given in online Appendix A.

Proton and helium CT scans were acquired using a rotating platform mounted between the trackers in a step‐and‐shoot mode from 180 projections, separated by 2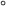 angular steps. Each projection was acquired from a single spill. Hence, each scan took a minimum of 24 min. While 90 projections at 4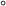 steps would be the usual procedure for stepped scans with the detector,^41^ this was shown to result in streak artifacts in HeCT scans.^24^, ^28^ On the other hand, taking even finer angular sampling was not feasible due to beam time constraints.

Prior to the scanning experiments, the energy detector response was calibrated. First, a run without any object placed between the tracker planes (empty run) was used to calibrate the dependence of the detector response on the lateral position of the incoming particles and to convert the detector's analog output to digital and then to energy as described in Bashkirov et al.^37^ Then, the energy response of the detector is calibrated to the water equivalent path length (WEPL) traversed by each particle using a calibration phantom of known RSP and geometry. Here, we followed the procedure detailed as configuration C in Piersimoni et al.^42^: A projection of a polystyrene wedge phantom RSP was acquired, which provides a smooth thickness variation between 50.8  and 0 mm. Then, adding one‐by‐one four polystyrene bricks of 50.8 mm thickness to the setup enabled to calibrate the dynamic WET range of the detector.

Initial filters acting on the reconstructed track segments and the energy loss in the five‐stage energy detector were applied to particle data to remove nuclear interactions and particles that scattered outside the detector.^43^ For the helium scans, a δE‐E technique was used to identify and remove secondary fragments for helium ion imaging.^28^ Although the filter showed to also be capable of removing nuclear interaction contamination in a previous simulation study on pCT,^44^ we did not apply it for the pCT results in this work as it resulted in a slight reduction of RSP accuracy. Secondary 3σ filters acting on the angular displacement (difference between incoming and outgoing particle angle) and WEPL of the particles were applied to remove remaining particle histories with unusual WEPL and those that underwent large angle scattering events.^43^ For filtering, the particles were binned into a grid of 1 mm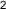 pixels according to the midpoint position of a straight line path connecting their entrance and exit point of the reconstruction volume.

Images were reconstructed using the DROP‐TVS iterative reconstruction algorithm,^45^ a block iterative algebraic reconstruction method applied in combination with the superiorization methodology using the total variation as the target function. A Feldkamp–David–Kress reconstruction assuming straight particle paths was used as the initial iterate for the DROP‐TVS algorithm and to detect the object's hull for particle path estimation.^46^ Within the DROP‐TVS algorithm, particle paths were estimated using the phenomenological cubic spline path formalism developed by Collins‐Fekete et al.^47^ that was shown to be also valid for helium ions.^48^ For the iterative reconstruction, the particle histories were divided into 40 blocks according to the previous implementation of the DROP‐TVS algorithm,^45^ each providing an updated estimate of the RSP map used as a starting point for the next block iteration. The relaxation parameter of the iterative solver was set to λ=0.1. The DROP‐TVS algorithm was stopped after 25 iterations to ensure algorithmic convergence. Of note, the DROP‐TVS algorithm has been employed with the same prototype pCT detector in several past studies,^24^, ^28^, ^32^, ^41^, ^42^, ^44^ yielding high quality pCT images even if only a limited number of projections is available.^41^ The pixel size was set to 0.976 mm and the slice thickness was 2 mm, to be consistent with the SECT/DECT scans.

### 2.6 Data analysis and uncertainty estimation

For each imaging modality, we obtained maps of voxelwise RSP estimates. From these RSP maps, we estimated an average RSP and standard deviation per sample by measuring the RSPs in a volume of interest (VOI) within the cylinder. The VOIs are cylindrical and were chosen to enclose the 2σ width of the carbon beam used for the reference measurement, resulting in a radius of 2 σ = 4.8 mm and VOIs of *V* = 2660 mm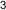. For each tissue sample and imaging modality, we hence obtained an average RSP per sample RSP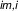, where im = (SECT, DECT, pCT, HeCT) and i=(1,2,…17) represents the index of the tissue and water samples. Additionally, we obtain the standard deviation of the RSP estimates, $\sigma_{im,i}$, and the standard error of the mean, calculated as

(4) $$\sigma_{\bar{im,i}}=\frac{\sigma_{im,i}}{\sqrt{N_{\text{vox}}}},$$

with 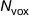 the number of voxels in the VOI. The average RSPs measured in these VOIs was used for comparison against the reference RSP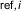, acquired using the peak detection measurement as detailed in Section 2.3. For each sample and imaging modality, we calculated the relative difference to the reference RSP using

(5) $$\Delta {\text{RSP}}_{i}=\frac{{\text{RSP}}_{im,i}-{\text{RSP}}_{\text{ref},i}}{{\text{RSP}}_{\text{ref},i}}.$$

To obtain an overall uncertainty of the RSP difference, we used error propagation to combine the uncertainty from the peak detection measurement, that is, the uncertainty of the reference RSP originating from the heterogeneity of the sample $\sigma_{\text{ref},i}$, and the standard error of the mean from imaging modalities, $\sigma_{\bar{im,i}}$. Per sample and imaging modality, we calculated the combined uncertainty $\sigma_{tot,i}$ of the RSP difference using the laws of error propagation

(6) $$\sigma_{tot,i}=\sqrt{{\left(\frac{1}{{\text{RSP}}_{\text{ref},i}}\right)}^{2}\sigma_{\bar{im,i}}^{2}+{\left(\frac{{\text{RSP}}_{im,i}}{{\text{RSP}}_{\text{ref},i}^{2}}\right)}^{2}\sigma_{\text{ref},i}^{2}}.$$

The uncertainty over all tissues was estimated using

(7) $$\sigma_{\mathrm{tot}}=\frac{1}{N}\sqrt{\sum_{i=1}^{N}\sigma_{\bar{tot,i}}^{2}},$$

with the number of samples N=17. This way, we obtain an uncertainty that includes uncertainties on the reference measurement, heterogeneity of the sample, and uncertainty from imaging modality.

## 3 RESULTS

Figure 5a shows a slice of the PMMA cylindrical phantom containing the cylindrical containers with the fresh tissue samples. The slice contains voxelwise RSP values derived from the four investigated imaging modalities. Visually comparing SECT and DECT (top panel), we observe that DECT‐predicted RSPs are affected by image noise more severely than SECT, confirmed by the profiles across the phantom displayed in Figure 5b. Comparing pCT and HeCT (bottom panel), we observe that pCT images show ring artifacts to the extent of ∼1% variation around the mean. While not as pronounced, similar ring artifacts are present in the HeCT image, pointed out by the arrows in Figure 5b.

**FIGURE 5:**
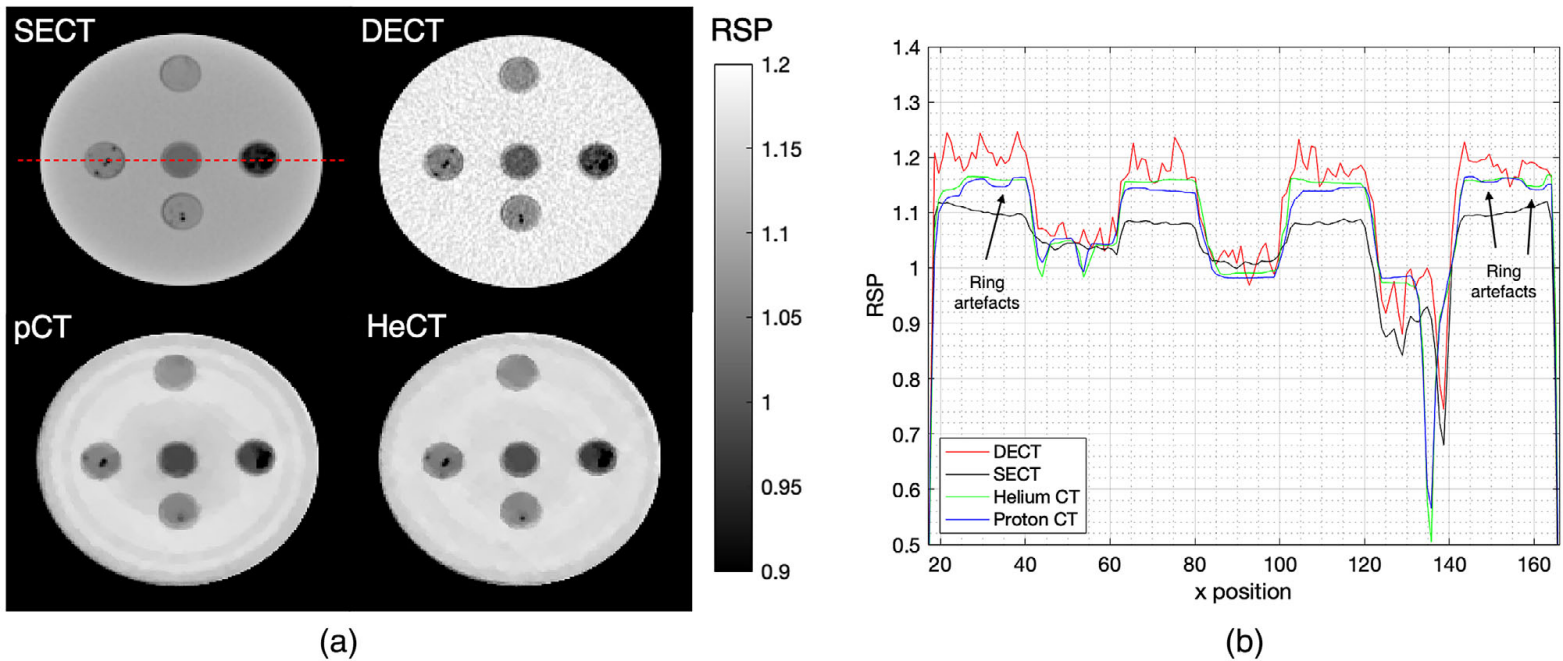
(a) Example tomographic slice of the cylindrical PMMA phantom with porcine and bovine tissues sample for each of the four modalities investigated in this work (SECT, DECT, pCT, and HeCT). (b) X‐profiles across the middle of the RSP maps. The position of the profile is indicated in (a). Of note, the increased noise in DECT and the ring artifacts in proton CT reconstructions (indicated by arrows) are known features.

Table 3, first column, shows the reference RSP values with measurement uncertainties determined using Equation (3). The following columns show the percent difference between reference RSP and p redicted RSP of each investigated CT modality (standard deviation), standard error of the mean, and combined uncertainty from reference measurement and respective CT modality. It should be noted that the uncertainties and in particular the standard deviations given in this paper depend on the imaging dose. For both photon CT modalities, the CTDI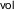 was kept equal for direct comparison. For particle CT, the lowest possible dose within the given delivery limitations was chosen, while still achieving a uniform fluence across the lateral field. No effort was made in this paper to achieve similar dose levels for all imaging modalities.

**TABLE 3:** Reference RSP and percentage difference to the reference RSP of the RSP predictions from SECT, DECT, pCT, and HeCT

		% Difference to reference (standard deviation)											
Tissue	RSP_ref_	SECT	$\sigma_{\bar{SECT}}$	$\sigma_{\mathrm{tot}}$	DECT	$\sigma_{\bar{DECT}}$	$\sigma_{\mathrm{tot}}$	pCT	$\sigma_{\bar{pCT}}$	$\sigma_{\mathrm{tot}}$	HeCT	$\sigma_{\bar{HeCT}}$	$\sigma_{\mathrm{tot}}$
Lung	0.90 (0.06)	3.01 (9.03)	0.25	6.47	0.32 (8.90)	0.24	6.30	1.74 (4.64)	0.13	6.39	0.29 (5.89)	0.16	6.30
Marrow	0.93 (0.04)	−3.44 (4.84)	0.13	4.05	1.11 (6.05)	0.16	4.24	1.80 (2.45) 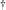	0.07	4.27	1.34 (2.75) 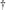	0.07	4.25
Back fat	0.97 (0.02)	−1.75 (1.74)	0.05	1.93	1.74 (3.10) 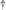	0.08	2.00	0.88 (0.72)	0.02	1.98	0.17 (0.75)	0.02	1.97
Belly fat	1.00 (0.01)	−4.57 (3.30) 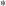	0.09	1.40	−0.24 (4.20)	0.12	1.46	−1.41 (1.27)	0.03	1.44	−0.76 (1.49)	0.04	1.45
Water	1.00 (0.00)	2.32 (0.31)	0.01	0.4	0.99 (2.30)	0.07	0.40	−1.50 (0.98)	0.04	0.38	−0.27 (0.33)	0.01	0.39
Brain	1.04 (0.01)	0.61 (0.84)	0.02	0.76	−0.43 (2.20)	0.06	0.75	0.08 (0.68)	0.02	0.75	−0.63 (0.80)	0.02	0.75
Kidney 2	1.04 (0.01)	−0.58 (5.07)	0.16	1.43	−1.09 (6.50) 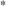	0.20	1.43	−0.16 (3.22)	0.12	1.44	−1.20 (4.24)	0.16	1.42
Kidney 1	1.05 (0.01)	−0.20 (3.40)	0.09	1.00	−0.37 (4.01)	0.11	1.00	−0.89 (1.20)	0.03	0.99	−0.95 (1.54)	0.04	0.99
Blood	1.05 (0.01)	0.43 (0.29)	0.01	0.53	0.51 (2.03)	0.06	0.54	1.15 (0.36)	0.01	0.54	0.72 (0.40)	0.01	0.53
Cheek muscle	1.05 (0.02)	−0.72 (1.43)	0.04	1.58	0.22 (3.05)	0.08	1.60	−1.33 (1.15)	0.03	1.57	−0.47 (1.39)	0.04	1.59
Leg muscle	1.05 (0.02)	−0.14 (1.59)	0.04	1.78	−0.50 (2.82)	0.08	1.78	0.71 (0.76)	0.02	1.80	0.19 (1.20)	0.03	1.79
Loin 1	1.06 (0.01)	−0.20 (2.27)	0.06	1.24	0.47 (3.76)	0.10	1.25	−1.73 (1.22) 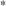	0.03	1.22	−0.78 (1.80)	0.05	1.23
Loin 2	1.06 (0.01)	−0.77 (1.55)	0.05	1.22	−0.49 (2.93)	0.09	1.22	−1.26 (1.03)	0.04	1.21	−1.23 (1.17) 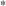	0.04	1.21
Liver 1	1.06 (0.01)	−0.75 (4.88)	0.13	1.15	−1.01 (5.53)	0.15	1.15	−0.85 (1.48)	0.04	1.15	−0.85 (1.82)	0.05	1.15
Liver 2	1.06 (0.01)	−0.80 (6.68)	0.21	1.07	−0.69 (7.31)	0.23	1.08	−1.05 (5.67)	0.21	1.07	−1.10 (6.46)	0.24	1.08
Trabecular bone	1.19 (0.08)	6.52 (4.84)	0.13	7.38	−0.19 (4.90)	0.13	6.91	−0.45 (4.56)	0.12	6.89	0.48 (4.66)	0.12	6.96
Cortical bone	1.78 (0.05)	8.22 (8.53) 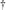	0.23	2.79	−0.07 (8.23)	0.22	2.58	−1.28 (2.62)	0.07	2.54	−0.17 (2.59)	0.07	2.57
Mean error	–	**0.42 (4.58)**	**0.03**	**0.70**	**0.02 (5.19)**	**0.03**	**0.68**	−**0.32 (3.43)**	**0.02**	**0.68**	−**0.30 (3.69)**	**0.02**	**0.68**
Mean absolute error	–	**2.06**	**0.03**	**0.70**	**0.61**	**0.03**	**0.68**	**1.07**	**0.02**	**0.68**	**0.68**	**0.02**	**0.68**
Root mean square error	–	**3.10**	**0.13**	**2.88**	**0.75**	**0.14**	**2.80**	**1.19**	**0.08**	**2.81**	**0.78**	**0.09**	**2.81**

Notes: For each imaging modality, the minimum and maximum prediction errors of the corresponding modality are indicated by the symbols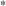 and 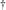, respectively. Standard deviations from each measurement are quoted in parenthesis, the standard error of the mean $\sigma_{\bar{x}}$ is quoted for each imaging modality. The total uncertainty $\sigma_{\mathrm{tot}}$ is the combined uncertainty from reference measurement (representing tissue heterogeneity) and imaging modality (SEM). The horizontal lines separate different groups of tissue (low‐density region, soft tissue region, bone tissue region) similar to Figure 6.

To demonstrate statistical significance of the results, we performed two‐sided *t*‐tests to compare the RSP distributions measured for each tissue with each imaging modality, with results summarized in online Appendix B. With few exceptions, the measured RSPs from the imaging modalities are significantly different from each other. For both liver samples, however, the significance test indicated that the RSP samples were taken from distributions of equal mean and variance. Percent differences of all imaging modalities are illustrated in Figure 6 for direct comparison.

**FIGURE 6:**
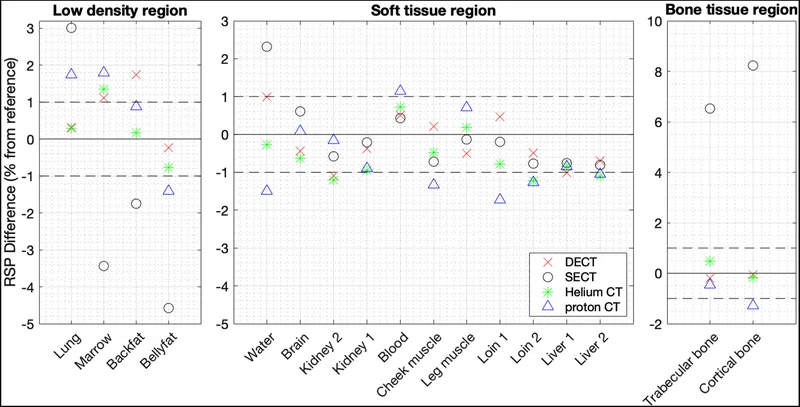
Percent RSP difference from the reference RSP for each imaging modality.

For SECT, we observe RSP prediction errors (standard deviation) between −4.57 ±1.40% in belly fat and 8.22 ±2.79% in cortical bone. Figure 6 shows that while SECT predicts well the soft tissue RSPs; high prediction errors occur in lower density adipose tissues and higher density bone tissues. DECT reduces the RSP inaccuracies observed with SECT and predicts most investigated tissues well within 1%, with errors between −1.09 ± 1.43% in kidney tissue and 1.74 ±2.00% in back fat. It should be noted that quantitatively, DECT presents the highest level of noise, observed in the standard deviations of the individual tissues and the standard error of the mean (0.87% over all tissues).

For pCT, we observe RSP errors between −1.73±1.22% in loin muscle and 1.80±4.27% in bone marrow. For HeCT, we observe RSP errors between −1.23 (1.21)% in loin and 1.34±4.25% in bone marrow. Both particle CT modalities have considerably lower levels of standard deviations for individual tissue RSPs and combined uncertainty of the mean error, attributed to the lower noise levels in the particle CT images. Besides, on average, pCT and HeCT exhibit a systematic underestimation of the RSP, which may be explained by imperfect calibration of the energy detector response.

Over all tissues, we find that DECT has the smallest RSP prediction error with a mean error (ME) of 0.02±0.68%, a mean absolute error (MAE) of 0.61% and a root mean square error (RMSE) of 0.75%. Those results are closely followed by HeCT, which shows a ME of −0.30 ±0.68% and comparable MAE (0.68%) and RMSE (0.78%). pCT presents a ME of −0.32 ±0.68%, MAE and a RMSE of 1.07% and 1.19%, respectively, with RSP accuracy affected by the ring artifacts also observed in Figure 5. SECT has the highest prediction errors with a ME of 0.42±0.70%, MAE of 2.06% and a RMSE of 3.10%. For comparison, the average RSP values and standard deviations measured in the bulk plastic part of the phantom are 1.09 ± 0.003% for SECT, 1.18±0.028% for DECT, 1.16± 0.005% for pCT, and 1.16±0.003% for HeCT. As observed in Figure 5, SECT underestimates the RSP of the bulk material by 6.0%, while DECT, though noisier, overestimates it by 1.7%. The misrepresentation of RSP values of plastic materials in photon CT is attributed to the use of calibration curves optimized for biological tissues.

## 4 DISCUSSION

The purpose of this work was to compare four systems representing four different imaging modalities capable of producing RSP maps for treatment planning in particle therapy. While each of the system was previously investigated individually for its capacity to predict RSPs, no overall comparison using fresh tissues has been presented so far. In this paper, we used a set of 16 fresh tissue samples to compare the RSP accuracy of SECT, DECT, pCT, and HeCT. We predominantly used porcine tissues that were processed minimally (not frozen, ground, etc.) and can be assumed approximately representative of human tissues. Of note, the lung sample was deflated and is therefore presenting with a higher density than a living lung. We found that DECT and HeCT can estimate RSPs over all tissues with an RMSE of 0.75 ± 2.80% and 0.78 ±2.81%, respectively, and pCT with an RMSE of 1.19±2.81%. The lowest accuracy is found in SECT with an RMSE of 3.10 ± 2.88%.

As it is the first time that fresh tissue RSPs were compared against a reference measurement for pCT/HECT, it is hard to evaluate our results against other groups. Still, DeJongh et al.^49^ conducted a relative comparison between the reconstructed RSP values for pCT and SECT using two heterogeneous porcine tissue samples with pCT and SECT. They observed an agreement between SECT and pCT in soft tissues better than 1%, whereas in bone tissues they reported relative differences of up to 6.9%. This observation is corroborated by our study. In soft tissues, SECT yielded an uncertainty similar to the other imaging modalities, whereas in bone tissue and adipose, SECT performed worse. We observed differences of 3.8% between the mean reconstructed RSP in belly fat with HeCT and SECT, and even 8.4% in cortical bone. A comparison of the RSP accuracy achievable in plastic samples to that in tissues is limited, due to the different nature of the experiments, with one being much more controlled than the other. Nevertheless, the here observed results for HeCT are also comparable to a previous study from our group investigating the RSP accuracy of HeCT for seven plastic materials,^28^ where a MAE of 0.45% was found. Similarly, our pCT results can be compared to recent experiments with the detector at the Chicago Proton Center,^50^ where a MAE of 0.87±0.03% was reported for the same plastic materials before applying an empirical artifact correction method. Given the respective uncertainties, no statistically significant difference between the results reported in our work and these experiments can be concluded.

Still, there are aspects of the current particle imaging system which leave room for further improvements. Ring artifacts were present in the reconstructed images that negatively affect RSP accuracy. These were more severe for pCT than for HeCT and comparable in magnitude to pCT scans from other centers.^20^ These artifacts result from the longitudinal segmentation of the detector which causes ambiguities in the calibration procedure.^20^, ^37^ Due to the dead material between scintillator stages and the noise threshold applied to the scintillator output, there is some ambiguity for particles stopping near one of the stage interfaces of the multistage detector leading to increased errors.^37^ The difference in ring artifacts between helium/proton is attributed to the particle range related to that energy threshold, that is the length traveled by a 1‐MeV particle corresponds to a smaller residual range for helium ions compared to protons, potentially causing the observed reduction of ring artifacts. Another source of uncertainty in our pCT images may be due to the more unstable particle rate used for protons compared to the helium ion rate used in this work. This limitation was imposed by the sensitivity limitations of the beam current monitoring system at the HIT facility experimental room nozzle. The response of the energy detector comprising scintillator stages read out by photomultiplier tubes is correlated to the particle rate. Calibration of the PMT gain and noise pedestal were performed for each projection using particles that did not cross the phantom, but a strongly fluctuating particle rate may lead to a broadening of the pedestal and gain distribution, and, hence, to a less precise baseline for the calibration.

It has to be noted that the δE‐E filter for removing secondary particles in the energy detector was applied to HeCT but not to pCT in this work, despite a previous simulation study from our group^44^ reporting improved pCT accuracy from 0.55% MAE without filter to 0.19% MAE with filter. For the experimental scans presented in this work, the δE‐E filter removed a similar nuclear interaction background as in the simulation, but this produced a slight decrease in overall RSP accuracy from 1.07% MAE without filter to 1.18% with filter. This may be related to detector effects not considered in the simulation work which are not yet fully understood. Hence, we kept the data processing for pCT to the conventional procedure with the detector.^43^ A more detailed technical investigation on the increased uncertainty for pCT compared to HeCT with this detector is planned.

It is worth mentioning that both HeCT and pCT showed a similar systematic underestimation of the RSP accuracy, which has also been observed already by Dedes et al.,^20^ hinting at a potential bias due to the detector calibration or the reconstruction procedure. Similar problems have been observed in the recent work by Dickmann et al.^50^ Using a new calibration phantom, they developed an empirical correction method which improved the accuracy of their experimental pCT results by reducing ring artifacts in the reconstructions. Using their method, they demonstrated a systematic bias of −1.3% in the reconstructed RSP values before applying the empirical artifact correction. Assuming that this work was affected by a similar bias, with the correction method, the particle CT RSP accuracy may be improved beyond that of DECT.

Finally, the presented RSP results for pCT and HeCT are also subject to the chosen reconstruction algorithm. The DROP‐TVS iterative reconstruction method we applied has been broadly employed with the used pCT prototype.^24^, ^28^, ^32^, ^41^, ^42^, ^44^ Especially, it has been shown to be able to provide high‐quality images acquired from a low number of projections.^41^ On the other hand, being an iterative reconstruction method the outcome is influenced by the chosen reconstruction parameters, especially the number of iterations. In that regard, we terminated the algorithm after 25 iterations, as this provided high RSP accuracy at a still comparatively low noise. Higher number of iterations increased mean RSP accuracy, but also increased the image noise.

It is important to note that we are not considering the statistical noise intrinsically which would be a measure of intrapixel variation, but rather a combination of inter–intra pixel noise due to the statistical correlation between adjacent pixel in the reconstruction.^51^ Still, this noise variation represents the expected noise in treatment planning and is the metric of interest here.

Our SECT/DECT results are very much in‐line with what can be found in recent literature. For comparison, we found that SECT could provide RSP measurement with a MAE(RMSE) of 2.06(3.10)% whereas DECT would provide 0.61(0.75)%. Bär et al.^16^ analyzed the capacity of DECT to extract WET in heterogeneous tissue samples, from which they extracted RSP values. They found a RSP RMSE of 0.61% for DECT and 1.59% for SECT. These measurements are, however, averaged over a thick phantom, which may explain the lower SECT RMSE error. Möhler et al.^17^ compared DECT/SECT‐predicted RSP for 65 bovines samples and found that SECT could predict RSPs of all tissues with a mean absolute error of (1.27 ± 0.12)% whereas DECT yielded a mean absolute error of (0.10 ± 0.15)%; a number lower than what was found in our study. However, their sample holder had a volume of 15 × 17.8 × 17.8 mm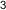 (4.75 cm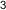) compared to our acquisition volume of 15.72 cm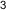. Larger heterogeneities were likely introduced within this larger piece of tissue. Further studies investigating animal tissues were presented by Taasti et al.^18^ and Xie et al.,^19^ with results comparable to this study.

We found that DECT‐predicted RSP maps are noisier compared to SECT‐predicted RSP maps, which we observe qualitatively in the images and quantitatively in the higher standard deviations. Several reasons for increased noise levels can be pointed out, such as the choice of reconstruction kernel and a reduced number of photons per DECT image compared to SECT acquisition. In addition to those factors, however, it appears that the nature of DECT prediction methods, which are often using polynomial fits to obtain the desired quantities such as RED, EAN, and RSP, is amplifying image noise in the parametric maps.^15^, ^52^ The parametric maps inferred from SECT are less affected by noise since the bi‐linear calibration is not so sensitive to small variations in the CT numbers. While a simple CT number to RSP lookup approach is advantageous in the presence of noise, it also suggests a poor sensitivity to resolve small variations in tissue densities and RSPs, leading to poor parametric accuracy as observed in our SECT results. Simard et al.^31^ thoroughly investigated RSP noise related to DECT, focusing on the technique proposed by Bourque et al., that we also used in our work. Using 13 homogeneous tissue‐mimicking plastics (Gammex RMI 467 inserts), they found an overall standard deviation on the RSP predictions of 4.21%, attributed to the use of least squares optimization techniques to estimate the parametric maps. Simard et al. adapted the method by Bourque et al. to a maximum a posteriori framework instead of the least squares method. With this framework, they were able to reduce the standard deviation on RSP predictions to 2.18% in the homogeneous tissues, a noise reduction by a factor of 1.9, while maintaining the RSP accuracy. Applied to the standard deviation found in this study, the standard deviation over all tissues of 5.19% on DECT‐predicted RSP would be reduced to 2.73%.

One important factor when comparing the different imaging modalities is the dose to the patient. While we could not measure the dose given for pCT and HeCT directly, we refer to a very similar study based on a TOPAS Monte Carlo simulation of the US pCT collaboration prototype detector.^24^ Based on this study, the total dose delivered for the presented HeCT can be extrapolated to be ∼6.6 mGy, while that for pCT was ∼4.7 mGy, presenting a potential dose advantage over SECT/DECT of a factor 9 and a factor 13, respectively. It has to be noted that the applied particle fluence was not optimized but rather set to the minimum threshold from beam delivery constraints. In this scenario, electronic noise and other acquisition related noise might dominate, and the dose might therefore be further lowered without significant loss in image quality. This is especially the case for pCT, where the operation at a higher particle rate than the intended 1.2 MHz limit of the system led to a decreased detection efficiency, which would not be the case at other centers. For example, Dedes et al.^20^ report pCT images with good plastic material RSP accuracy at a delivered dose below 2 mGy.

In this work, each of the systems suffers from limitations and offers certain benefits for treatment planning. SECT does not yield very accurate RSP maps, and DECT does so at the cost of increased noise (see Figure 5). In addition, both methods suffer from beam hardening artifacts (notably seen in the SECT scan in Figure 5), and their respective RSP maps are acquired usually days before the treatment in a different setup (couch and patient position), thus potentially introducing positioning and registration uncertainties. On the other hand, they are widely available for clinical usage with proven technology. pCT provides images in situ immediately before the treatment, using the same beam, positioning and couch as for treatment, thereby minimizing registration uncertainties. However, it yields lower spatial resolution (roughly 5 lp/cm at 200 MeV/u for a 20‐cm phantom^25^, ^53^) that may underestimate range mixing effect. HeCT will reconstruct sharper images (up to 10 lp/cm at 200 MeV/u for a 20‐cm phantom, comparable to clinical photon CT resolution^24^, ^29^, ^54^, ^55^), but requires a heavy ion synchrotron system operating at the required energy to penetrate the patient, which is a rare modality in the field of particle therapy. When considering the currently available RSP acquisition methods for clinical usage, considerations must be given not only to the accuracy but also to the acquisition time, the modality's availability, as well as the indirect uncertainties added by each respective method (e.g., registration). By factoring in the high accuracy achieved by DECT (matched only by HeCT), its widespread usage (with clinical implementation currently underway), and its speed of acquisition, we find that this modality emerges as the clear choice from this study, especially if it is built into the treatment room. SECT, which is currently still the most used modality for treatment planning and verification, appears as the worst choice of the four modalities explored here. pCT and HeCT are more ambivalent; they can produce higher RSP map accuracy in situ but need more developments to be clinically usable. It is necessary that correction methods, such as the one proposed by Dickmann et al.,^50^ are implemented for the ring artifacts we observed with the current pCT prototype, which should not be necessary for future clinical pCT systems. In addition, significant reduction of acquisition time for single event particle imaging by about one order of magnitude compared to the current benchmarks (∼6 min for a full scan at a cyclotron and with continuous data acquisition^38^) is required for particle CT to become clinically relevant.

## 5 CONCLUSION

We performed a complete comparison of state‐of‐the‐art SECT and DECT with a prototype pCT and HeCT to predict RSP maps for the use in particle therapy treatment planning. Overall, DECT and HeCT provided the highest overall RSP accuracy well below 1%. The novel modalities pCT and HeCT have the potential for further improvements in terms of image quality (ring artifacts) and acquisition speed. Proton and helium ion imaging have further promising applications (e.g., dynamic tracking, patient positioning) that will be crucial for developing the next generation of high‐precision particle therapy treatment.

## CONFLICT OF INTEREST

The authors have no conflicts of interest to disclose.

## Supporting information

Supporting Information.

## Data Availability

The data that support the findings of this study are available from the corresponding author upon reasonable request.
