# Supplementary material for: Experimental comparison of photon versus particle computed tomography to predict tissue relative stopping powers
Source: Med Phys. 2021 Nov 16;49(1):474–87. doi: 10.1002/mp.15283 (PMC13655494; doi:10.1002/mp.15283)
Supplement: Supplementary file 1 [file MP-49-474-s001.pdf]

Appendix A: X-ray spectra

Appendix B: Parameters for particle CT acquisition

Figure 1(a) shows the particle rate for an example run without phantom in the beam line for HeCT, figure 1(b) the equivalent for pCT. Shown are both the trigger rate (true particle rate) and the rate of events saved for output. The particle rate for pCT was less stable, and above the systems intended maximum particle rate of 1.2 MHz, resulting in reduced detection efficiency.

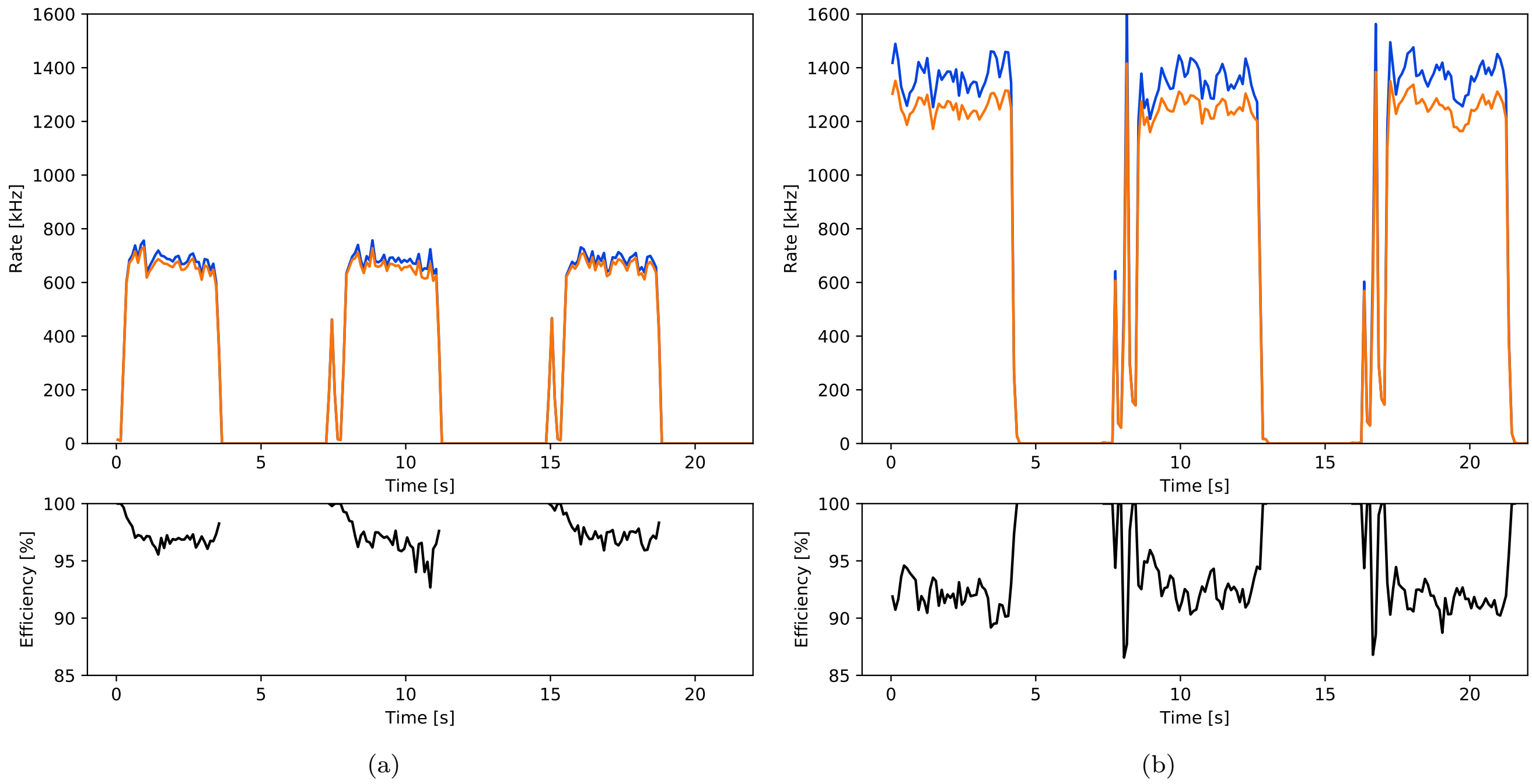

FIG. 1: Top: Spill intensity as measured by the detector triggers (blue), and corresponding rate of events saved for output (orange) for an example helium ion (a) and proton (b) run with no phantom in the beam line. Bottom: Relative difference of trigger and event rate, i.e., detection efficiency.

Figure 2 shows the lateral profile of event counts measured on the rear tracker of the pCT prototype for an example helium ion (top) and proton (bottom) projection (multiple spills) with no phantom in the beam line. Comparatively stronger fluctuations in the proton counts are seen, related to the higher and less stable particle rate. For the figures, only valid particle tracks were considered, i.e., events with multiple valid track options in the tracking detectors were removed.

Appendix C: Two-sided t-test

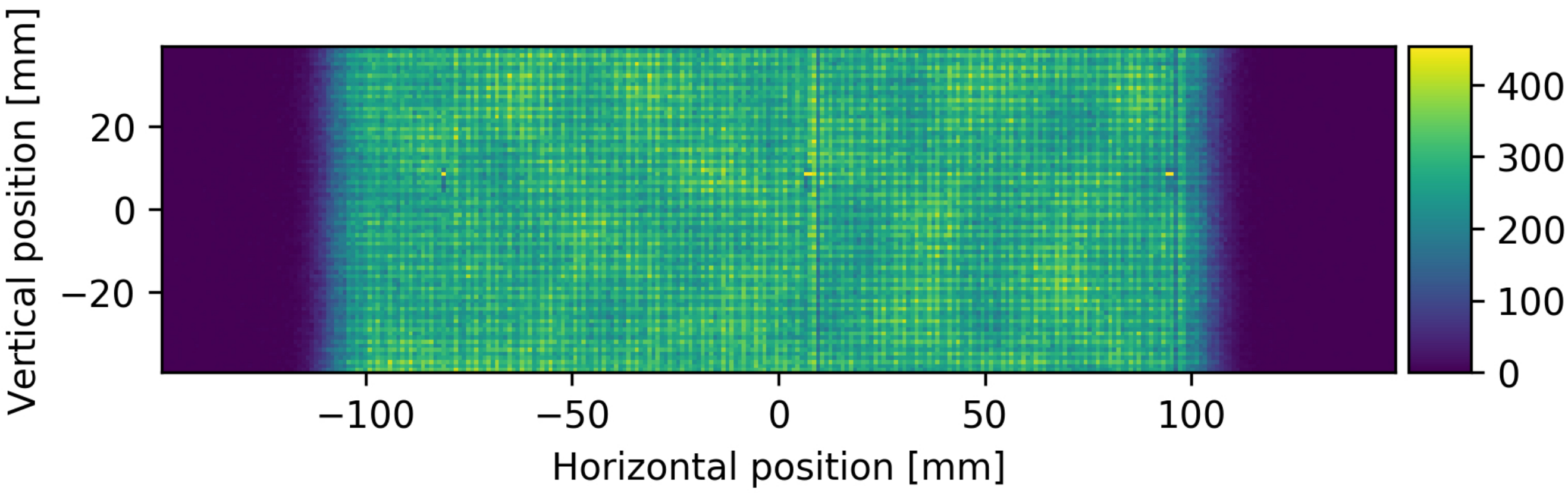

(a)

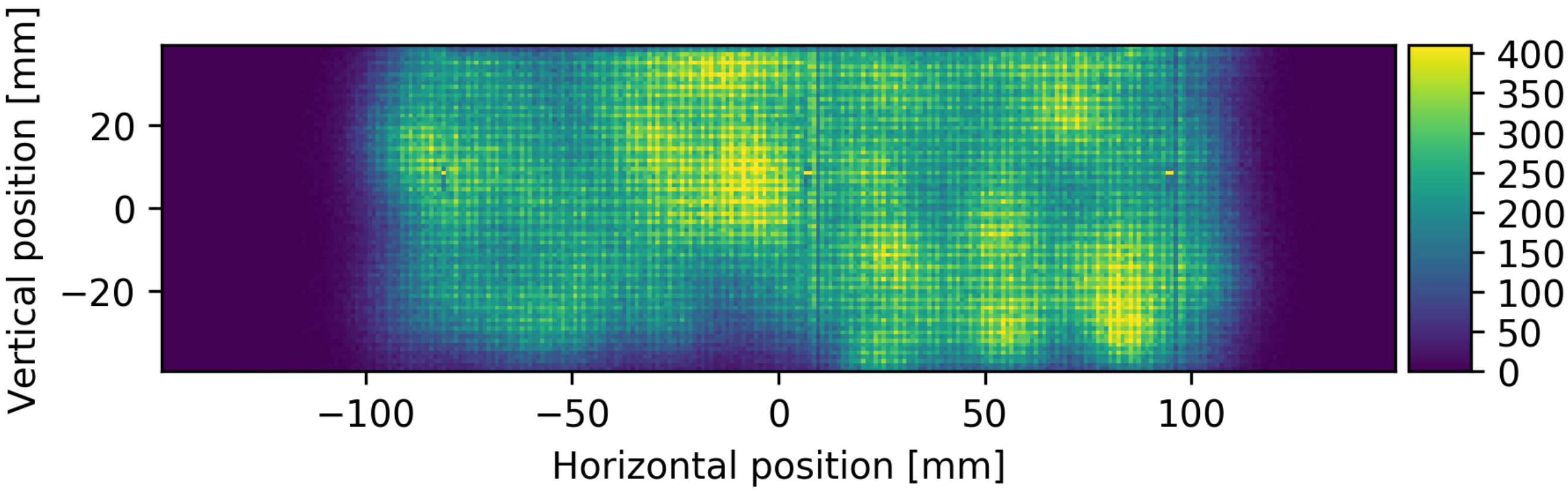

(b)

FIG. 2: Lateral profile of particle counts at the entrance of the energy detector given by the rear tracker measurement for an example helium ion (a) and proton (b) run with no phantom in the beam line. Tracker pile-up events were already rejected for the figure.

TABLE I: Results of the statistical significance analysis (two-sided t-test). Tested were the RSP distributions collected from each material and the four different imaging modalities. The null hypothesis is that the RSP distributions of two imaging modalities come from independent samples of normal distributions of equal means and variance. In the table, the decision h of the significance analysis using a significance level of 5% is displayed. If $h = 0$, the null hypothesis is accepted (p>0.05) and it can be assumed that the distributions have equal means and variances. If $h = 1$, the null hypothesis is rejected ($p < 0.05$).

Tissue	SECT/DECT	SECT/pCT	SECT/HeCT	DECT/pCT	DECT/HeCT	pCT/HeCT
Lung	1	1	1	1	0	1
Marrow	1	1	1	1	0	1
Back fat	1	1	1	1	1	1
Belly fat	1	1	1	1	1	1
Water	1	1	1	1	1	1
Brain	1	1	1	1	1	1
Kidney 2	1	1	1	1	0	1
Kidney 1	0	1	1	1	1	0
Blood	0	1	1	1	1	1
Cheek muscle	1	1	1	1	1	1
Leg muscle	1	1	1	1	1	1
Loin 1	1	1	1	1	1	1
Loin 2	1	1	1	1	1	0
Liver 1	0	0	0	0	0	0
Liver 2	0	0	0	0	0	0
Trabecular bone	1	1	1	0	1	1
Cortical bone	1	1	1	1	0	1
